# HIV Infection and Osteoarticular Tuberculosis: Strange Bedfellows

**DOI:** 10.1155/2016/5718423

**Published:** 2016-05-26

**Authors:** B. Hodkinson, N. Osman, S. Botha-Scheepers

**Affiliations:** ^1^Department of Medicine, Division of Rheumatology, University of Cape Town and Groote Schuur Hospital, Cape Town 7945, South Africa; ^2^Division of Anatomical Pathology, National Health Laboratory Service, University of Cape Town and Groote Schuur Hospital, Cape Town 7945, South Africa

## Abstract

We report the case of a 47-year-old female patient with rheumatoid arthritis and HIV infection presenting with a 3-week history of a painful swollen knee, increased serum inflammatory markers, and a low CD4 lymphocyte count. The diagnosis of TB arthritis was made by synovial fluid culture, GeneXpert/PCR, and confirmed by histopathology of a synovial biopsy. A mini literature review suggests that although HIV infection is associated with extrapulmonary TB, osteoarticular TB is a relatively unusual presentation in an HIV positive patient. The diagnostic utility of the GeneXpert test is explored. We also describe the patient's good response to an intra-articular corticosteroid injection in combination with standard anti-TB therapy.

## 1. Introduction

Patients infected with human immunodeficiency virus (HIV) are at increased risk of tuberculosis (TB) coinfection. We describe an unusual presentation of HIV and TB coinfection in a patient with rheumatoid arthritis (RA). We present a mini literature review and discussion regarding 3 aspects of this case:

- the association between HIV and osteoarticular TB;
- diagnostic tests;
- adjuvant management.

## 2. Case Presentation

A 47-year-old female with seropositive RA was diagnosed 6 years previously with a 3-week history of a painful right knee. She reported no fever, night sweats, cough, or loss of weight and had received no recent intra-articular steroid injections. Two years earlier she was diagnosed with HIV infection and was initiated on combination antiretroviral (ARV) therapy (tenofovir/efavirenz/emtricitabine). Her RA therapy consisted of methotrexate (MTX) 15 mg weekly and prednisone 7.5 mg daily and the RA had been in remission for 24 months prior to this presentation. She had no history of previous TB.

**Figure 1:**
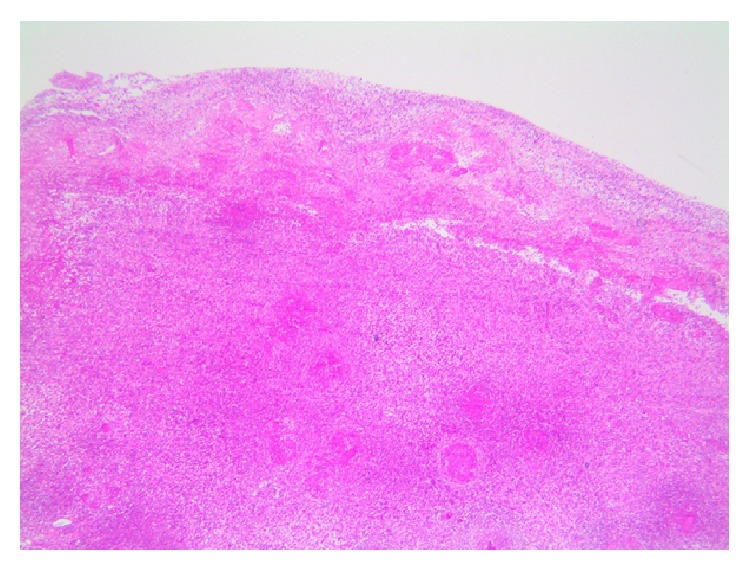
Haematoxylin and Eosin (H&E) stain of the synovial biopsy showing foci of granulomatous inflammation (20x magnification).

**Figure 2 fig2:**
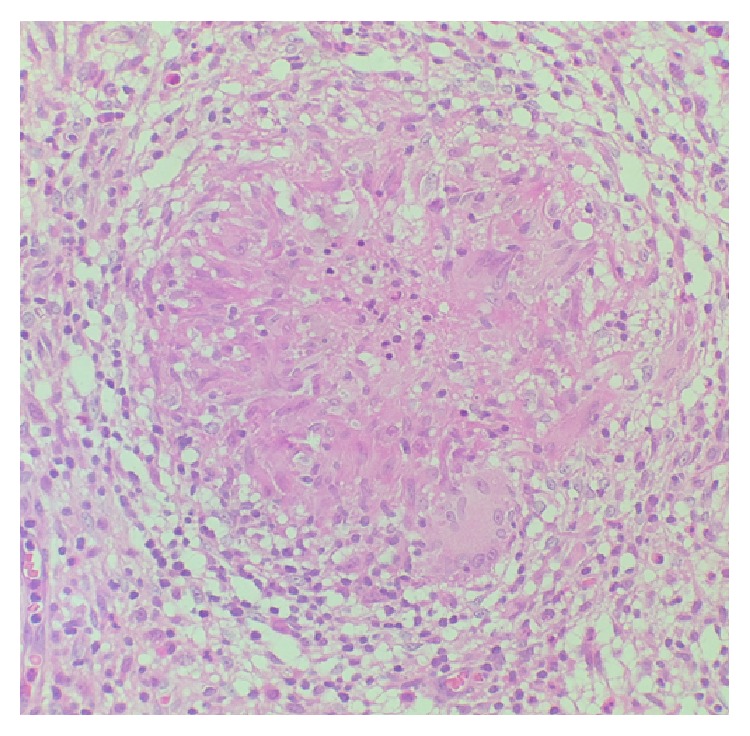
H + E stain of synovial biopsy showing granulomatous inflammation (400x magnification).

Examination revealed a thin middle-aged female with a swollen tender right knee with a reduced range of motion. She was afebrile with clinically inactive RA, and there was no tenderness or swelling of her other joints. Blood investigations showed a normochromic anaemia (hemoglobin = 10.3 g/dL), leukocytosis (WCC = 19.3 × 10^9^/L), and thrombocytosis (platelet = 486 × 10^9^/L). Her C-reactive protein was elevated (340 mg/L) and CD4 cell count was low (199/mm^3^). Chest and knee radiographs were normal. Pus was aspirated from the right knee and submitted for microbiology with Gram and Ziehl-Neelsen stain negative. GeneXpert on the fluid was positive. Culture was positive at 21 days for* Mycobacterium tuberculosis*. A synovial biopsy submitted for histology revealed necrotising granulomatous inflammation and the Ziehl-Neelsen stain was positive for acid-fast bacilli (Figures 1−3) confirming the diagnosis of osteoarticular TB/tubercular arthritis. MTX was discontinued, and combination antituberculous therapy was started. Due to the ongoing symptoms of the arthritis, a single intra-articular corticosteroid injection (methylprednisolone acetate 80 mg) was administered. Clinical improvement and a good range of motion of the right knee were noted within 2 weeks. At her follow-up visit at 3 months she was symptom-free and had recovered the full range of motion of the joint. Her blood counts normalised, her CRP was 3, and the CD4 cell count improved to 410/mm^3^.

## 3. Discussion

The single most important risk factor for contracting TB is HIV infection, particularly in Sub-Saharan Africa where HIV-related TB comprises 79% of TB cases [1]. In particular, extrapulmonary forms of TB (ExP-TB) are encountered in HIV positive patients with low CD4 lymphocyte counts. Skeletal TB is an uncommon form of ExP-TB and affects either the spine (comprising 5% of ExP-TB) or peripheral joints as “osteoarticular TB (OA-TB)” which constitutes a further 5% of ExP-TB. Usually presenting as a chronic monoarthritis, most often affecting the hip, knee, or wrist joint, OA-TB typically has an insidious onset without constitutional symptoms or features of pulmonary TB [2].

Many reports claim a strong association between OA-TB and HIV infection, including a review from Zambia that describes 60% of OA-TB cases as HIV-associated [3]. However, studies of OA-TB, including studies from areas of high HIV prevalence, report very few HIV positive cases (Table 1), suggesting that HIV infection may not be a risk factor for OA-TB. It has been shown in Soweto, South Africa, that although HIV is associated with ExP-TB, the relative frequency of OA-TB amongst HIV positive patients was significantly lower than in HIV negative patients [4]. A South African study of TB-associated skeletal changes revealed an increase in the number of cases with skeletal TB, from 28% in the pre-1985 era to 41% after 1985 [5]. In the case of TB spine, there may be more convincing evidence of an association with HIV, with 17–33% of cases testing HIV positive in areas with a high prevalence of HIV (such as South Africa, Nigeria, Morocco, and Spain) and a much lower incidence (0–8%) elsewhere [6]. A recent report from SA described 20 cases of TB spine with half of these patients coinfected with HIV [7]. HIV positive patients have been shown to have less vertebral body destruction and more abscess formation compared to HIV negative patients. Microscopic features between the two groups are similar but an inverted CD4 : CD8 lymphocyte ratio is seen within the granulomas of patients coinfected with HIV [8, 9].

The synovial fluid of our patient was GeneXpert positive. GeneXpert MTB/RIF (Cepheid, Sunnyvale, CA, USA) is a polymerase chain reaction (PCR) assay allowing rapid diagnosis of TB and detection of resistance to rifampicin. Sputum GeneXpert detects with a high specificity the majority of pulmonary TB cases and is a useful screening test for ExP-TB, in particular CSF and tissue specimens [10]. For the diagnosis of TB spine, PCR tests on pus or vertebral bone samples have a very high sensitivity and specificity (96% and 96–100%, resp.) [11, 12]. In the case of synovial fluid, PCR tests have shown moderate sensitivity (63%) but excellent specificity (92–100%) [13]. Thus PCR tests are a useful and very convenient test for OA-TB, but synovial biopsy should be performed in patients with a negative test. A recent study from Mexico demonstrated the excellent clinical utility of serum PCR in the diagnosis of spine and OA-TB, with sensitivity and specificity of 91% and 97%, respectively [14].

The patient described in this case study was on MTX, low dose oral corticosteroids, and ARV therapy. The safety of MTX in patients who are HIV positive is uncertain. In patients with low CD4 counts, MTX may predispose patients further to opportunistic infections, including TB [15]. The use of MTX in HIV positive individuals may be acceptable in the setting of a CD4 count >200, particularly if ARV therapies are prescribed and if the patient is closely followed up.

We treated our patient with antituberculous therapy and an intra-articular corticosteroid injection, with a fairly dramatic resolution of signs and symptoms and restoration of joint function. The rationale for this management approach was based on the postulated role of the immune-mediated response to TB in the development of joint damage in OA-TB. Reducing inflammation may preserve the articular cartilage and joint space. In a study of rabbits with staphylococcal septic arthritis, intra-articular steroids reduced joint damage [16]. A similar approach in humans may be beneficial [17]. In two randomized placebo-controlled trials in children with bacterial arthritis, adjuvant intravenous corticosteroids in combination with antibiotic therapy reduced the clinical symptoms and improved outcomes without any adverse effects [18, 19].

Adjuvant corticosteroids reduce complications and improve survival in TB meningitis and in the case of TB pericarditis reduce the incidence of constrictive pericarditis, which may be analogous to joint destruction and contractures in the setting of OA-TB [20, 21]. Randomized controlled studies would establish the role of adjuvant intra-articular corticosteroids in the management of OA-TB.

In summary, this case is a relatively unusual presentation of ExP-TB in an HIV positive patient. The diagnosis was aided by a positive GeneXpert test, and an excellent outcome was achieved. Adjuvant intra-articular corticosteroids may have hastened resolution of the clinical symptoms and signs of infection.

## Figures and Tables

**Figure 3:**
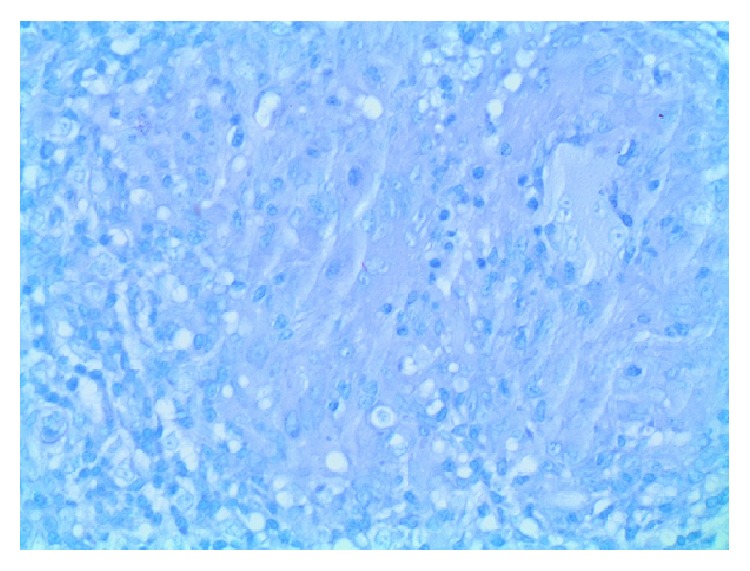
Ziehl-Neelsen stain of synovial biopsy showing acid-fast bacillus (arrow) (400x magnification).

**Table 1:** Case series of osteoarticular TB with HIV status.

Country	Duration of study	Number of HIV+ cases/no of OA-TB^*∗*^ cases (%)	Antiretroviral therapy
India [22]	2010–2012	0/13	—
India [23]	—	0/93	—
Thailand [24]	1997–2006	1/77	—
Nigeria [25]	1998–2009	0/97^*∗∗*^	
France [26] (including 74% African immigrants)	1980–1994	1/206^*∗∗*^	
United Kingdom [27] (including 89% South Asian immigrants)	1988–2005	0/44	—
Denmark [28] (including 50% Somalian refugees)	1993–1997	3/26^*∗∗*^	—
US [29]	1999–2003	1/31 (3.2%)	All on ARV therapy
China [30]	2011-2012	0/43	—
Thailand [31]	1994–2002	1/27 (3.7%)	—

OA-TB: osteoarticular TB; ARV therapy: antiretroviral therapy.

^*∗*^Extraspinal OA-TB cases only.

^*∗∗*^Spine and extraspinal OA-TB cases combined.
